# Retraction Note: m^6^A modification-mediated CBX8 induction regulates stemness and chemosensitivity of colon cancer via upregulation of LGR5

**DOI:** 10.1186/s12943-023-01874-z

**Published:** 2023-10-06

**Authors:** Yi Zhang, Min Kang, Bin Zhang, Fanchao Meng, Jun Song, Hiroshi Kaneko, Fumio Shimamoto, Bo Tang

**Affiliations:** 1 Department of Health Sciences, Hiroshima Shudo University, 1-1-1, Ozuka-higashi, Asaminami-ku, Hiroshima, 731-3195 Japan; 2 Department of General Surgery, Affiliated hospital of Xuzhou Medical University, Xuzhou, 221000 China; 3 Department of Obstetrics, Gynecology and Reproductive Sciences, Yale School of Medicine, New Haven, CT 06510 USA; 4 Department of Oncology, The First Affiliated Hospital of Dalian Medical University, Dalian, 116011 China

**Retraction note:** ***Mol Cancer*** **18, 185 (2019)**

10.1186/s12943-019-1116-x

The Editor in Chief has retracted this article after concerns were raised about potential image overlap in Figs. 1 and 6. Therefore, the Editor has lost confidence in the data presented here. None of the authors has responded to any correspondence from the editor/publisher about this retraction.

- Partial image overlap between Fig. 1A specifically the upper image for shCtrl with Fig. 1J specifically the upper image for LGR5+.
- Image overlap between Fig. 1H specifically cells at position 2 in 3rd lane for shCBX8-2 with Fig. 2G specifically cells at position 1 in 5th lane for vec + CPT-11.
- Image overlap between Fig. 6F specifically the last lane of WB for ß-actin with Fig. 5A of [1].
- Image overlap between Fig. 1I specifically the last lane for shCBX8-2 with Fig. 4H of [2].
- Image overlap between Fig. 1I specifically the last lane for shCBX8-2 with Fig. 8A of [3].
- Image overlap between Fig. 1I specifically the last lane for shCBX8-2 with Fig. 8A of [4].
